# User-Centered Virtual Reality for Promoting Relaxation: An Innovative Approach

**DOI:** 10.3389/fpsyg.2019.00479

**Published:** 2019-03-12

**Authors:** Silvia Francesca Maria Pizzoli, Ketti Mazzocco, Stefano Triberti, Dario Monzani, Mariano Luis Alcañiz Raya, Gabriella Pravettoni

**Affiliations:** ^1^Department of Oncology and Hemato-Oncology, University of Milan, Milan, Italy; ^2^Applied Research Division for Cognitive and Psychological Science, European Institute of Oncology IRCCS, Milan, Italy; ^3^Institute of Research and Innovation in Bioengineering, Universidad Politécnica de Valencia, Valencia, Spain

**Keywords:** virtual reality, relaxation, emotion regulation, personalized virtual reality, user-centered virtual reality

## Abstract

Virtual reality has been used effectively to promote relaxation and reduce stress. It is possible to find two main approaches to achieve such aims across the literature. The first one is focused on generic environments filled with relaxing “narratives” to induce control over one’s own body and physiological response, while the second one engages the user in virtual reality-mediated activities to empower his/her own abilities to regulate emotion. The scope of the present contribution is to extend the discourse on VR use to promote relaxation, by proposing a third approach. This would be based on VR with personalized content, based on user research to identify important life events. As a second step, distinctive features of such events may be rendered with symbols, activities or other virtual environments contents. According to literature, it is possible that such an approach would obtain more sophisticated and long-lasting relaxation in users. The present contribution explores this innovative theoretical proposal and its potential applications within future research and interventions.

## Introduction

Virtual reality (VR) has been successfully employed to promote relaxation and reduce stress, and it has notably matured trough time, showing a relevant potential in improving and regulating emotional well-being.

Two main approaches aiming at achieving relaxation, stress reduction and emotion regulation can be found in the literature. The first one employs mainly “generic environments” by which users are exposed to relaxing narratives stimuli and try to gain control over body physiological activation, while the second one requires users to be active, implying an interaction with VR contents to train emotion regulation.

However, in the epoch of personalization a new approach is needed that provides the user with a more adequate and person-adapted techniques. In the present contribution a third approach is therefore proposed, which integrates and extends those mentioned above, but is based on its specific methodological assumptions.

This third perspective would propose a VR based on personalized contents, built on distinctive features picked-up by users’ memories of relevant life-events, and on adaptive virtual reality. The present contribution presents and discusses this novel approach and its similarities and dissimilarities with the previous ones, highlighting how personalized contents features can be identified and their possible applications and benefits in the field of relaxation and emotion regulation.

To trace similarities and differences between these 3 approaches, a list of features was built up (Table 1), trying to describe their specific and common characteristics.

**Table 1:** Characteristics of the three main approaches to virtual reality for promoting relaxation or emotion regulation.

Attributes	(1) approach: Relaxing VR	(2) approach: Engaging VR	(3) approach: Personalized VR
Psychological or physiological target	Relaxation	emotion regulation, stress management training	Relaxation, emotion regulation, stress management training, well-being empowerment
Aim	Transient relaxation	Empower and train users’ abilities	Transient relaxation and/or empower and train users’ abilities
Contents	Generic scenarios and stimuli (often nature-based)	Specific scenarios and interactive stimuli to promote training	User-centered contents
Users’ involvement	Passive	Active	Passive or Active
Contents and stimuli	Fixed	Based on emotion to induce or ability to train	Adaptive (i.e., modifiable in real time basing on users’ current state)
Technology	Virtual reality, psychophysiological correlates	Virtual reality, psychophysiological correlates, gaming/interaction	Virtual reality, psychophysiological correlates, gaming, Artificial Intelligence


## The First Approach: Relaxing VR

The first approach, which we will name “relaxing VR” (rVR henceforth), presents contents inspired or directly derived from classical relaxation techniques such as progressive muscle relaxation, autogenic training, yoga, meditation; typically, the user is shown environments that can help him/her feel safe. Often, virtual environments feature contents that are generically associated with pleasant, peaceful, non-arousing sceneries such as islands, parks, gardens and other open-space, generic nature-based environments. Indeed, as often used in imagery techniques too, these environments have proved to be a valuable means to reduce stress (Baños et al., 2005; León-Pizarro et al., 2007; Villani et al., 2007; Felix et al., 2017), both in healthy and pathological contests (e.g., pain) (Hoffman et al., 2011). Within the rVR approach, natural scenarios and visual or auditory natural elements have been frequently employed, showing a fair efficacy in several contexts (Annerstedt et al., 2013; Anderson et al., 2017). For example, regarding sound features specifically, nature-based VR scenarios filled with natural sounds resulted in being more effective for stress reduction, compared with natural scenarios without them (Annerstedt et al., 2013). These rVR interventions usually present users with multimodal stimuli, involving visual, auditory and haptic modalities to try lowering physiological activation, gaining control over body reactions. Thus, not only visual natural elements, but also auditory natural elements display some intrinsic relaxing properties (Saadatmand et al., 2013). Apart from natural sounds, rVR scenarios present auditory stimuli that may include warm and calm voices, giving instructions to relax muscles, relieve stress and negative thoughts. Controlling breathe frequency and amplitude is another technique usually delivered by rVR narratives, in that breathing exercises are widely employed in clinical psychology (Smith, 1999).

Relaxing VR is a useful application of VR because relaxation states, by lowering general arousal, are proven to have positive effects on cognitive and physical stress. The main aim of this first approach is to put users in a more or less passive state of relaxation, or to lower physiological arousal, inducing a positive state of well-being. This is the case not only of relaxation-focused interventions, but also of those interventions aiming at induce positive mood states, through the exposition to emotionally connoted scenarios (Baños et al., 2004, 2008; Riva et al., 2007).

Importantly, such interventions induce a transitory state of relaxation or positive emotion (Felnhofer et al., 2015). Users may come even to a deep relaxation state, but such physiological state and its benefits are usually not maintained for long in the everyday life and the VR experience could hardly be generalized to other contexts. Indeed, the purpose of rVR is to reach a positive state of well-being in the very moment of the VR exposure, rather than engage users in a learning process to gain new abilities. That’s not to say that rVR cannot produce long-lasting positive effects, as relaxation or positive emotions can produce even medium and long term benefits on well-being, coping strategies and health (Folkman and Moskowitz, 2000; Fredrickson, 2001; Tugade et al., 2004; Lyubomirsky et al., 2005; Pressman and Cohen, 2005; Cohn et al., 2009).

## The Second Approach: Engaging VR

Targeting a learning process to empower users is in fact one of the core differences between rVR and the second approach that can be found in the literature, which we will label engaging VR (eVR henceforth).

Under the term “eVR,” we classify those interventions that try to build learning processes about one’s own emotional and behavioral abilities, giving users a flexible and modifiable environment. This kind of approach does not merely imply a passive visualization of the virtual environment or exposure to relaxing stimuli, rather it requires users to interact with virtual contents, permitting the acquisition of specific skills. It is the case of emotion regulation training in VR and of some therapeutic interventions in VR.

Broadly, emotion regulation refers to how individuals influence and express the emotions they experience and how they experience them, and it has been defined as “all of the conscious and no conscious strategies we use to increase, maintain, or decrease one or more components of an emotional response” (Gross et al., 1998).

Virtual reality therapies have been employed for several psychopathological conditions: anxiety disorders (Baños et al., 2011), specific phobias (Parsons and Rizzo, 2008), eating disorders (Ferrer-García and Gutiérrez-Maldonado, 2012; Ferrer-García et al., 2013), trauma and stress-related disorders (Gonçalves et al., 2012), as well as other serious psychiatric conditions (Maples-Keller et al., 2017a,b). VR therapy interventions have some characteristics in common with eVR, as the virtual environments vary according to the symptoms to be addressed.

A customized environment is used also in “stress inoculation training,” which shares some similarities with VR therapies, and in particular with Virtual Reality Exposure Therapy (VRET). Based on the assumption that gradual exposure to fear-inducing stimuli can increase “mental readiness,” stress inoculation training can train subjects’ stress response capacity (Bosse et al., 2014).

Training emotional reactivity in response to negative stimuli has resulted to be a valuable primary prevention intervention for burn-out and psychological functioning for different work categories (Rizzo et al., 2008; Popović et al., 2009; Bosse et al., 2014) and it represents a case of eVR, as it aims to improve stress response capacity, targeting mental presence and emotional reactivity in stressful situations.

During the training, users are helped gain skills to cope with negative stimuli and feelings, managing the emotions they experience in a more functional way. By increasing emotional management, such interventions have an important impact on well-being and mental health functioning (Serino et al., 2014).

For example, job interviews have been simulated in virtual environments to give participants the opportunity to exercise their ability to manage emotions before and after a 5-weeks long emotional skills training (Villani et al., 2017). Also, VR applications have been used to train children with autism spectrum disorder to learn how to deal emotionally with everyday life social situations (Ip et al., 2018).

For the sake of completeness, it is interesting to note that innovative resources to improve/exercise emotional skills come from the video games scenario. Indeed, the association between gaming experiences and real life abilities improvements has been established (Lobel et al., 2014), with the capacity of video games to modulate arousal and interoceptive awareness. As highlighted by a recent review, commercial video games are valuable tools to improve emotion regulation capacities, training emotional intelligence and emotional strategies (Villani et al., 2018). Many emotional experiences can be practiced when playing video games, ranging from primary emotions such as fear, pleasure and anger (Rodríguez et al., 2015b; Wrzesien et al., 2015; Vara et al., 2016; Hemenover and Bowman, 2018), to complex emotions such as feeling “enriched” (Oliver et al., 2016) or feeling socially engaged with other characters. The first kind of emotions is more contingent and easily induced, through narratives and *ad hoc* scenarios, while the latter is usually evoked by complex narratives, requiring for example characters that have a long and struggling journey (Oliver et al., 2012).

Apart from entertainment, that of course is an emotional outcome of playing video games, players experience personally meaningful gaming experiences, encountering opportunities for introspection through the identification with characters and avatars (Oliver et al., 2016).

Importantly, rVR and eVR approaches are far from being mutually exclusive, on the contrary they can be used conjointly. It is the case for example of the VRET, that, as in the traditional psychotherapeutic settings from whom it is derived, requires the patient to be systematically desensitized (Marks and Gelder, 1965) within a combination of relaxation and exposure. The great majority of the VRET studies in fact combined the two approaches (Maples-Keller et al., 2017a), with an initial phase with psychoeducational methods and breathing or relaxing exercises. The combination of both interventions has to be preferred, as in the traditional techniques and relaxation training on its own for specific phobia has shown reduced efficacy compared to exposure intervention (Mühlberger et al., 2001).

## The Third Approach: Personalized VR

The third approach, “personalized VR” (pVR henceforth), would be different from the previous ones, specifically for what concerns the choice and the construction of VR contents and environments.

Personalized VR would be in fact a user-centered approach to the design and implementation of the VR setting itself.

A pVR would be defined by two main characteristics, one relating to the design of VR contents, and the other to the technology to implement. The design aspect regards **preliminary investigation about users’ relevant life events**, aiming to extract distinctive perceptive features of personal memories and experiences. If a relaxing environment has to be built, a preliminary inquiry about potential users’ relaxing life events will be conducted, while for an emotional training VR, user would be asked for emotionally connotated memories. Such descriptions should be accurate, carefully recorded and should include multisensory details: visual, tactile, auditory and even olfactory elements.

Description should include multisensory elements, both to have detailed elements and to understand with which perceptive modality the user re-experience the autobiographical scene (sounds, temperature, and colors). User would also be explicitly asked about which stimuli they associate with this experience, trying to find out significant perceptive cues effective in memories elicitation (Holland and Kensinger, 2010).

**FIGURE 1:**
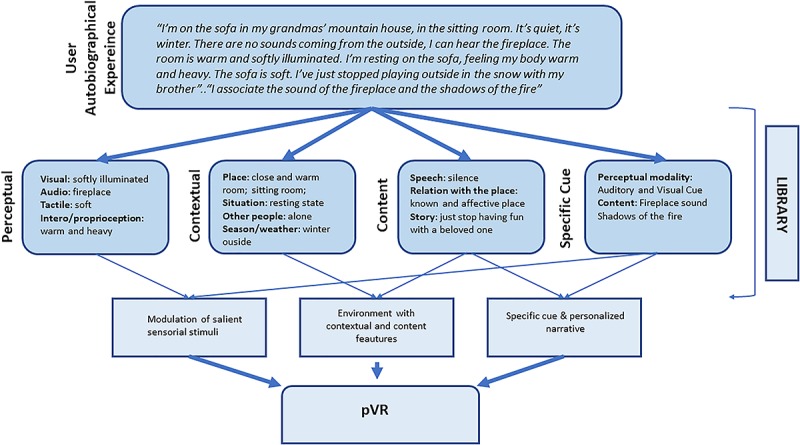
The extraction process of relevant information to build pVR, to inform the construction of personalized environments. Adopting a pVR approach does not mean to digitally re-create specific situations, but to understand which symbols/features could be digitally rendered in order to allow the participants recall ancient sensations.

As a second step, distinctive features of such autobiographical life events and evoking cues would be rendered with symbols, activities or other virtual environments contents, through a qualitative analysis of users’ descriptions (see Figure 1).

The pVR approach would target autobiographical memories both because it is important to show the user’s preferred places (Fisher, 1974; Kyle et al., 2004; Korpela et al., 2008; Korpela and Ylén, 2009), and because recalling personal experience can bring sensorial and vivid feelings (Rubin and Kozin, 1984; Tulving, 1985; Wright and Gaskell, 1992; Brewer, 1996; Rubin, 2005).

Not only user-centered approaches (e.g., based on users’ feedback for content) have proved to be particularly effective in different applications of VR (Parsons et al., 2009; Rizzo et al., 2011), but knowing which perceptive and contextual elements connote emotional memories can also help in emotion elicitation or emotion modulation, and can inform experimenters and developers on which elements have to be modulated or have not to be included, depending on the target of the intervention. pVR environments and stimuli would be built focused on the specific users’ characteristics and, more specifically, on the environments features users give importance and relevance to, eventually acting as a cue for recalling personal past memories and enhancing users involvement.

The vividness of emotional engagement grows when something personal, related to the subjective experience, is presented. Re-evoking personal contents in fact enhance affective psychological states (Picard et al., 2001; Witvliet et al., 2001), augmenting the capacity to elaborate memories and emotional experiences.

Previous studies have already proved that putting autobiographical stimuli within the VR environment helps give a vivid emotional connotation to the experience and enhance sense of presence (Baños et al., 2004, 2008, 2009; Rey et al., 2005; Riva et al., 2007).

In the above-mentioned studies, different emotional states (such as relaxation, joy, sadness), were evoked through autobiographical stimuli presented in different sensorial modalities (picture, audio, music, autobiographical recall), suggesting that autobiographical contents in VR may play a valuable role in subjective emotional experience. This might constitute a relevant difference from the rVR and eVR, which typically lack of contents tailored on their users.

pVR would address stress and emotion regulation, both relying on complex circuit involving cognitive, behavioral and psycho-physiological responses (Lang, 1968; Borkovec and Costello, 1993), targeting (a) a bottom-up modality with personalized stimuli and autobiographical cues and (b) addressing physiological correlates through adaptative methods. In a relaxing environment, element recalling previous safe place would enhance feelings of security and peace, while in an emotion regulation training, providing affective connotated stimuli could help emotion regulation and arousal modulation by giving the user the occasion to confront his/her own relevant life events (Holland and Kensinger, 2010).

Relaxation can be guided for example with verbal instructions bringing attention to muscles activation and breathing (Jacobsen, 1929; Miller, 1987), or induced through cognitive representations of positive thoughts and stimuli (Tusek and Cwynar, 2000; Vempati and Telles, 2002). pVR can be modified according to the relaxing technique employed in the specific pVR intervention, rather it would stress user-centered environment and autobiographical engagement (Holland and Kensinger, 2010) to enhance sense of presence and affective involvement. Furthermore, pVR would provide adaptative physiological mechanism, monitoring physiological parameters.

Differently from the studies employing unique autobiographical contents and stimuli, in which contents have to be specifically selected for each subject, the pVR approach would allow for a broader generalization, reached through the construction of a stimuli library, based on memories relevant features.

From a methodological point of view, user centered design techniques would be implemented as the foundation for the VR design. These techniques come from the user experience field, but they are not focused on evaluating technologies (e.g., in terms of usability and functionality), rather they are meant to be used *before* the tool or technology is created, in order to provide information to design in terms of users’ needs, intentions and contexts of use (Garrett, 2010; Triberti and Barello, 2016). On the one hand, techniques such as contextual inquiry (which is a field research method, a semi-ethnographic interview to be conducted within the place/context of interest) (Holtzblatt and Jones, 1993) could be a valuable tool to enrich users’ subjective testimony with objective measures and properties to be included in a personalized virtual environment; similarly, empathy maps, which are a visual diagram featuring user personas’ experience and needs (Curedale, 2016), could be used to systematize information on users to guide pVR implementation.

User Centered research could allow pVR to develop *trans*-situational knowledge about the most common or recurrent multi-sensorial features of relaxing experiences in order to develop a library of stimuli which can be used to approximate the personalized virtual scenario for each possible user.

The second main characteristic of the virtual tools created within a pVR approach, relating to technology to be implemented, is that of **adaptive virtual reality** (Alcañiz et al., 2007, 2009; Parsons and Reinebold, 2012): in order to be *really* personalized, a virtual environment should be able to adapt its own contents to the user’s state in the very moment of the interaction. In other words, pVR environments would feature integrated systems able to sense and analyze users’ state by means of psychophysiological correlates, self-reported states, and observable behaviors in order to provide modifications to the virtual experience itself. Doing so, pVR would not be “user centered” only in the sense that user’s pre-existing needs, experiences and memories are taken into consideration for design, but also personalized changes will happen within the virtual instance according to users’ current state.

As written above, relevant properties of personal memories would be organized in terms of macro and sub-categories (e.g., perceptual aspects such as form and color of objects; content aspect such as the meaning to be communicated through discourses present in the VR; etc.) and the system would operate such properties dynamically during any VR instance, in order to maintain a set level of immersion and emotional reaction by the user, continually monitored through psychophysiological indexes.

In the future, from a technological point of view, such adaptation feature of virtual reality would be exploited within the merging of VR and Artificial Intelligence (AI). AI techniques, such as machine learning, deep learning and natural language processing (NLP) provide computers with reasoning and analytical capabilities that, until quite recently, it was possible to achieve only with standalone servers in specialized laboratories. Today, any field of knowledge can employ AI capabilities due to the development of cloud-based AI servers. Historically, AI has been associated to virtual reality technology mainly for what regarded the implementation of virtual agents (Luck and Aylett, 2000). However, the implications of merging AI and VR for personalized VR experiences has been less studied. Research into VR focuses on computing techniques that bring humans with natural perceptual ways of interaction and new methods of thinking and learning in virtual and augmented environments. AI provides technologies that allow computers to mimic abilities that are exclusive to humans, such as intelligence and consciousness. These two fields have in common the enhancement of human abilities at a perceptual level and knowledge generation. The integration of both research fields will permit the development of more natural and realistic virtual environments in which humans and computers will interact naturally. The synergy between mixed reality interfaces, AI and the high-speed ubiquitous communication networks of the future will generate radically innovative human-human communication channels, with the intelligent processing of signals such as body movements, facial expressions, eye tracking, physiological variables and brain signals, among others. The majority of emerging technologies reports focus on three emerging technology mega-trends: AI everywhere, transparently immersive experiences and digital platforms (Gartner, 2017).

In pVR, such technology will be employed to analyze multiple, integrated data about VR users’ current state, to translate these into emotional information, and finally to modify the virtual stimuli. Recent studies (Rodríguez et al., 2015a; Bermudez i Badia et al., 2018; Marín-Morales et al., 2018) showed that it is possible to apply machine learning techniques to measure specific emotions in VR, so to extract a set of psychophysiological and behavioral features to support autonomous emotion recognition.

Four main phases are imagined for development of pVR:

(1) Employing correlational research and focused user-centered interviews to gather information on common features of people memories about meaningful relaxing/positive experiences;
(2) Creating multiple versions of virtual environments based on recurring categories about sensations, concepts, and symbols in participants’ testimony;
(3) Evaluating the adaptable virtual environments with a benchmarking approach, testing pVR products in comparison to already existing one, sharing common features with the pVR intervention of interest (aim, type of environment, adaptative or not, target users) (a) prior with already existing virtual scenarios in the field of relaxation or emotion training that can be find in commerce and (b) then the with those instruments that have been already scientifically tested;
(4) Developing adaptive virtual environments, based on the ability to analyze and exploit users’ current states to adapt virtual contents to one’s own present state.

## Conclusion

The approach proposed in the present contribution promises to be an innovative advancement in the field of VR to positively manipulate the emotional experience of people. It is important to consider that the pVR approach is still in its infancy, or better, here it has been outlined *in nuce* only. However, it is possible not only to identify methodological guidelines to develop it, but also to highlight its possible limitations; specifically, it is still not very clear how to identify participants to the user research who would give developers the most meaningful information to start understanding relax/emotions personal experiences. Another issue related to sampling is connected to the inherently “personalized” nature of the VR contents within the pVR approach: participants could possibly report inadequate or unusable memories; for example, patients with chronic conditions tend to remember negative experiences because of the salience effect (Renzi et al., 2016), which may make difficult for them to recall positive experiences before the onset of chronic pain or pathological stress. Secondarily, issues related to pVR implementation still need to be taken into consideration, such as its cost compared to other approaches. We speculate that pVR could bring efficacy advantages, compared to non-personalized approaches, despite its major costs, in particular in the field of clinical psychology interventions, where a more general personalization of contents (for example in the case of specific phobias and VRET) is already applied to embrace users specificity. Overall, personalized approaches and personalized medicine bring economic advantages, focusing on personalized needs and on effective interventions for specific patients (Annemans et al., 2013). From an organizational point of view, being based on complex and possibly long-lasting user research, the pVR approach could be difficult to include within organizational practices (e.g., hospitals or other care facilities which employ VR for rehabilitation); indeed, its influences on practices by health professionals are not easy to prefigure and could generate risky courses of actions (Fairbanks and Wears, 2008; Gilardi et al., 2014).

While future technological advances in VR and AI promise to enable richer user experiences, perhaps the greatest revolution that will be made possible by next-generation pVR involves the ability to collect, analyze and make use of unprecedented amounts of data, that is, bringing pVR into the age of “Big Data Psychology” (Mitroff et al., 2015).

When the use of pVR sites become a commodity, there will be a large potential user base for pVR applications able to analyze huge sample sizes. The adaptation of A/B testing methods to pVR would give researchers the tools and the sample size to investigate the impact on patients of even minor changes in pVR. The combination of big data machine learning algorithms, capable of automatically identifying behavioral patterns and statistically predicting outcomes, will also continuously increase pVR efficacy. This analysis need not only be at a coarse level, aggregating data from millions of users, but might also be done at an individual level, for example, by learning which specific types of stimuli evoke a better patient experience in a particular user, and tailoring future scenarios accordingly.

Future research is needed to explore both the user research as main information guiding the design of pVR contents, and to test the efficacy of the first prototypes.

## Author Contributions

SP conceived the work and wrote the first draft. KM contributed in conceiving the work and helped to refine the theoretical framework. ST and DM performed the literature search and contributed with writing to the final version. MAR contributed with important intellectual content and helped to refine the theoretical framework. GP supervised the whole process and contributed with important intellectual content. All authors contributed to manuscript revision, read and approved the submitted version.

## Conflict of Interest Statement

The authors declare that the research was conducted in the absence of any commercial or financial relationships that could be construed as a potential conflict of interest.

## References

[B1] Alcañiz M. Botella C. Baños R. M. Zaragoza I. Guixeres J. (2009). The intelligent e-therapy system: a new paradigm for telepsychology and cybertherapy. *Br. J. Guid. Couns.* 37 287–296. 10.1080/03069880902957015

[B2] Alcañiz M. Botella C. Rey B. Baños R. Lozano J. A. de la Vega N. L. (2007). “EMMA: an adaptive display for virtual therapy,” in *Proceedings of the 3rd International Conference on Foundations of Augmented Cognition* (Berlin: Springer), 258–265. 10.1007/978-3-540-73216-7_29

[B3] Anderson A. P. Mayer M. D. Fellows A. M. Cowan D. R. Hegel M. T. Buckey J. C. (2017). Relaxation with immersive natural scenes presented using virtual reality. *Aerosp. Med. Hum. Perform.* 88 520–526. 10.3357/AMHP.4747.2017 28539139

[B4] Annemans L. Redekop K. Payne K. (2013). Current methodological issues in the economic assessment of personalized medicine. *Value Health* 16 S20–S26. 10.1016/j.jval.2013.06.008 24034308

[B5] Annerstedt M. Jönsson P. Wallergård M. Johansson G. Karlson B. Grahn P. (2013). Inducing physiological stress recovery with sounds of nature in a virtual reality forest — results from a pilot study. *Physiol. Behav.* 118 240–250. 10.1016/j.physbeh.2013.05.023 23688947

[B6] Baños R. M. Botella C. Alcañiz M. Liaño V. Guerrero B. Rey B. (2004). Immersion and emotion: their impact on the sense of presence. *Cyberpsychol. Behav.* 7 734–741. 10.1089/cpb.2004.7.734 15687809

[B7] Baños R. M. Botella C. Guerrero B. Liaño V. Alcañiz M. Rey B. (2005). The third pole of the sense of presence: comparing virtual and imagery spaces. *Psychnol. J.* 3 90–100. 10.1089/cpb.2008.0056 19018695

[B8] Baños R. M. Botella C. Guillen V. García-Palacios A. Quero S. Bretón-López J. (2009). An adaptive display to treat stress-related disorders: EMMA’s world. *Br. J. Guid. Couns.* 37 347–356. 10.1080/03069880902957064

[B9] Baños R. M. Botella C. Rubió I. Quero S. García-Palacios A. Alcañiz M. (2008). Presence and emotions in virtual environments: the influence of stereoscopy. *Cyberpsychol. Behav.* 11 1–8. 10.1089/cpb.2007.9936 18275306

[B10] Baños R. M. Guillen V. Quero S. Garcia-Palacios A. Alcaniz M. Botella C. (2011). A virtual reality system for the treatment of stress-related disorders: a preliminary analysis of efficacy compared to a standard cognitive behavioral program. *Int. J. Hum. Comput. Stud.* 69 602–613. 10.1016/j.ijhcs.2011.06.002

[B11] Bermudez i Badia S. Veléz Quintero L. E. Cameirao M. S. Chirico A. Triberti S. (2018). Towards emotionally-adaptive virtual reality for mental health applications. *IEEE J. Biomed. Health Inform.* 10.1109/JBHI.2018.2878846 [Epub ahead of print]. 30387752

[B12] Borkovec T. D. Costello E. (1993). Efficacy of applied relaxation and cognitive-behavioral therapy in the treatment of generalized anxiety disorder. *J. Consult. Clin. Psychol.* 61 611–619. 10.1037/0022-006X.61.4.611 8370856

[B13] Bosse T. Gerritsen C. De Man J. Treur J. (2014). Towards virtual training of emotion regulation. *Brain Inform.* 1 27–37. 10.1007/s40708-014-0004-9 27747526 PMC4883154

[B14] Brewer W. F. (1996). “What is recollective memory?,” in *Remembering Our Past: Studies in Autobiographical Memory*, ed. Rubin D. C. (New York, NY: Cambridge University Press), 19–66. 10.1017/CBO9780511527913.002

[B15] Cohn M. A. Fredrickson B. L. Brown S. L. Mikels J. A. Conway A. M. (2009). Happiness unpacked: positive emotions increase life satisfaction by building resilience. *Emotion* 9 361–368. 10.1037/a0015952 19485613 PMC3126102

[B16] Curedale R. (2016). *Empathy Maps: Stand in Your Customer’s Shoes.* Topanga, CA: Design Community College Incorporated.

[B17] Fairbanks R. J. Wears R. L. (2008). Hazards with medical devices: the role of design. *Ann. Emerg. Med.* 52 519–521. 10.1016/j.annemergmed.2008.07.008 18722693

[B18] Felix M. M. D. S. Ferreira M. B. G. da Cruz L. F. Barbosa M. H. (2017). Relaxation therapy with guided imagery for postoperative pain management: an integrative review. *Pain Manag. Nurs.* 10.1016/j.pmn.2017.10.014 [Epub ahead of print]. 29249618

[B19] Felnhofer A. Kothgassner O. D. Schmidt M. Heinzle A. K. Beutl L. Hlavacs H. (2015). Is virtual reality emotionally arousing? Investigating five emotion inducing virtual park scenarios. *Int. J. Hum. Comput. Stud.* 82 48–56. 10.1016/j.ijhcs.2015.05.004

[B20] Ferrer-García M. Gutiérrez-Maldonado J. (2012). The use of virtual reality in the study, assessment, and treatment of body image in eating disorders and nonclinical samples: a review of the literature. *Body Image* 9 1–11. 10.1016/j.bodyim.2011.10.001 22119329

[B21] Ferrer-García M. Gutiérrez-Maldonado J. Riva G. (2013). Virtual reality based treatments in eating disorders and obesity: a review. *J. Contemp. Psychother.* 43 207–221. 10.1007/s10879-013-9240-1

[B22] Fisher J. D. (1974). Situation-specific variables as determinants of perceived environmental aesthetic quality and perceived crowdedness. *J. Res. Pers.* 8 177–188. 10.1016/0092-6566(74)90019-1

[B23] Folkman S. Moskowitz J. T. (2000). Positive affect and the other side of coping. *Am. Psychol.* 55 647–654. 10.1037/0003-066X.55.6.647 10892207

[B24] Fredrickson B. L. (2001). The role of positive emotions in positive psychology. The broaden-and-build theory of positive emotions. *Am. Psychol.* 56 218–226. 10.1037/0003-066X.56.3.218 11315248 PMC3122271

[B25] Garrett J. J. (2010). *The Elements of User Experience: User-Centered Design for the Web and Beyond.* London: Pearson Education.

[B26] Gartner (2017). Available at: https://www.gartner.com/en/newsroom/press-releases/2017-08-15-gartner-identifies-three-megatrends-that-will-drive-digital-business-into-the-next-decade [accessed October 23, 2018].

[B27] Gilardi S. Guglielmetti C. Pravettoni G. (2014). Interprofessional team dynamics and information flow management in emergency departments. *J. Adv. Nurs.* 70 1299–1309. 10.1111/jan.12284 24138152

[B28] Gonçalves R. Pedrozo A. L. Coutinho E. S. F. Figueira I. Ventura P. (2012). Efficacy of virtual reality exposure therapy in the treatment of PTSD: a systematic review. *PLoS One* 7:e48469. 10.1371/journal.pone.0048469 23300515 PMC3531396

[B29] Gross J. J. Feldman Barrett L. John O. Lane R. Larsen R. Pennebaker J. (1998). The emerging field of emotion regulation: an integrative review. *Rev. Gen. Psychol.* 2 271–299. 10.1037/1089-2680.2.3.271

[B30] Hemenover S. H. Bowman N. D. (2018). Video games, emotion, and emotion regulation: expanding the scope. *Ann. Int. Commun. Assoc.* 42 125–143. 10.1080/23808985.2018.1442239

[B31] Hoffman H. G. Chambers G. T. Meyer W. J. III Arceneaux L. L. Russell W. J. Seibel E. J. (2011). Virtual reality as an adjunctive non-pharmacologic analgesic for acute burn pain during medical procedures. *Ann. Behav. Med.* 41 183–191. 10.1007/s12160-010-9248-7 21264690 PMC4465767

[B32] Holland A. C. Kensinger E. A. (2010). Emotion and autobiographical memory. *Phys. Life Rev.* 7 88–131. 10.1016/j.plrev.2010.01.006 20374933 PMC2852439

[B33] Holtzblatt K. Jones S. (1993). “Contextual inquiry: a participatory technique for system design,” in *Participatory Design: Principles and Practices*, eds Schuler D. Namioka A. (Hillsdale, NJ: Lawrence Earlbaum Publishers),177–210.

[B34] Ip H. H. S. Wong S. W. L. Chan D. F. Y. Byrne J. Li C. Yuan V. S. N. (2018). Enhance emotional and social adaptation skills for children with autism spectrum disorder: a virtual reality enabled approach. *Comput. Educ.* 117 1–15. 10.1016/J.COMPEDU.2017.09.010

[B35] Jacobsen E. (1929). *Progressive Relaxation.* Oxford: University of Chicago Press.

[B36] Korpela K. M. Ylén M. Tyrväinen L. Silvennoinen H. (2008). Determinants of restorative experiences in everyday favorite places. *Health Place* 14 636–652. 10.1016/j.healthplace.2007.10.008 18037332

[B37] Korpela K. M. Ylén M. P. (2009). Effectiveness of favorite-place prescriptions. *Am. J. Prev. Med.* 36 435–438. 10.1016/j.amepre.2009.01.022 19269127

[B38] Kyle G. Graefe A. Manning R. Bacon J. (2004). Effects of place attachment on users’ perceptions of social and environmental conditions in a natural setting. *J. Environ. Psychol.* 24 213–225. 10.1016/J.JENVP.2003.12.006

[B39] Lang P. J. (1968). “Fear reduction and fear behavior: problems in treating a construct,” in *Research in Psychotherapy*, ed. Shlien J. M. (Chicago, IL: American Psychological Association), 90–102.

[B40] León-Pizarro C. Gich I. Barthe E. Rovirosa A. Farrús B. Casas F. (2007). A randomized trial of the effect of training in relaxation and guided imagery techniques in improving psychological and quality-of-life indices for gynecologic and breast brachytherapy patients. *Psychooncology* 16 971–979. 10.1002/pon.1171 17311247

[B41] Lobel A. Granic I. Engels R. C. M. E. (2014). Stressful gaming, interoceptive awareness, and emotion regulation tendencies: a novel approach. *Cyberpsychol. Behav. Soc. Netw.* 17 222–227. 10.1089/cyber.2013.0296 24256133

[B42] Luck M. Aylett R. (2000). Applying artificial intelligence to virtual reality: intelligent virtual environments. *Appl. Artif. Intell.* 14 3–32. 10.1080/088395100117142

[B43] Lyubomirsky S. King L. Diener E. (2005). The benefits of frequent positive affect: does happiness lead to success? *Psychol. Bull.* 131 803–855. 10.1037/0033-2909.131.6.803 16351326

[B44] Maples-Keller J. L. Bunnell B. E. Kim S.-J. Rothbaum B. O. (2017a). The use of virtual reality technology in the treatment of anxiety and other psychiatric disorders. *Harv. Rev. Psychiatry* 25 103–113. 10.1097/HRP.0000000000000138 28475502 PMC5421394

[B45] Maples-Keller J. L. Yasinski C. Manjin N. Rothbaum B. O. (2017b). Virtual reality-enhanced extinction of phobias and post-traumatic stress. *Neurotherapeutics* 14 554–563. 10.1007/s13311-017-0534-y 28512692 PMC5509629

[B46] Marín-Morales J. Higuera-Trujillo J. L. Greco A. Guixeres J. Llinares C. Scilingo E. P. (2018). Affective computing in virtual reality: emotion recognition from brain and heartbeat dynamics using wearable sensors. *Sci. Rep.* 8:13657. 10.1038/s41598-018-32063-4 30209261 PMC6135750

[B47] Marks I. M. Gelder M. G. (1965). A controlled retrospective study of behaviour therapy in phobic patients. *Br. J. Psychiatry* 111 561–573. 10.1192/bjp.111.476.561 14344917

[B48] Miller K. M. (1987). Deep breathing relaxation. *AORN J.* 45 484–488. 10.1016/S0001-2092(07)68361-6 3548586

[B49] Mitroff S. R. Biggs A. T. Adamo S. H. Dowd E. W. Winkle J. Clark K. (2015). What can 1 billion trials tell us about visual search? *J. Exp. Psychol. Hum. Percept. Perform.* 41 1–5. 10.1037/xhp0000012 25485661

[B50] Mühlberger A. Herrmann M. J. Wiedemann G. C. Ellgring H. Pauli P. (2001). Repeated exposure of flight phobics to flights in virtual reality. *Behav. Res. Ther.* 39 1033–1050. 10.1016/S0005-7967(00)00076-0 11520010

[B51] Oliver M. B. Bowman N. D. Woolley J. K. Rogers R. Sherrick B. I. Chung M.-Y. (2016). Video games as meaningful entertainment experiences. *Psychol. Pop. Media Cult.* 5 390–405. 10.1037/ppm0000066

[B52] Oliver M. B. Hartmann T. Woolley J. K. (2012). Elevation in response to entertainment portrayals of moral virtue. *Hum. Commun. Res.* 38 360–378. 10.1111/j.1468-2958.2012.01427.x

[B53] Parsons T. D. Reinebold J. L. (2012). Adaptive virtual environments for neuropsychological assessment in serious games. *IEEE Trans. Consum. Electron.* 58 197–204. 10.1109/TCE.2012.6227413

[B54] Parsons T. D. Rizzo A. A. (2008). Affective outcomes of virtual reality exposure therapy for anxiety and specific phobias: a meta-analysis. *J. Behav. Ther. Exp. Psychiatry* 39 250–261. 10.1016/j.jbtep.2007.07.007 17720136

[B55] Parsons T. D. Rizzo A. A. Rogers S. York P. (2009). Virtual reality in paediatric rehabilitation: a review. *Dev. Neurorehabil.* 12 224–238. 10.1080/17518420902991719 19842822

[B56] Picard R. W. Vyzas E. Healey J. (2001). Toward machine emotional intelligence: analysis of affective physiological state. *IEEE Trans. Pattern Anal. Mach. Intell.* 23 1175–1191. 10.1109/34.954607

[B57] Popović S. Horvat M. Kukolja D. Dropuljić B. Cosić K. (2009). Stress inoculation training supported by physiology-driven adaptive virtual reality stimulation. *Stud. Health Technol. Inform.* 144 50–54. 19592729

[B58] Pressman S. D. Cohen S. (2005). Does positive affect influence health? *Psychol. Bull.* 131 925–971. 10.1037/0033-2909.131.6.925 16351329

[B59] Renzi C. Riva S. Masiero M. Pravettoni G. (2016). The choice dilemma in chronic hematological conditions: why choosing is not only a medical issue? A psycho-cognitive perspective. *Crit. Rev. Oncol. Hematol.* 99 134–140. 10.1016/j.critrevonc.2015.12.010 26762858

[B60] Rey B. Montesa J. Raya M. A. Baños R. M. Botella C. (2005). A Preliminary study on the use of an adaptive display for the treatment of emotional disorders. *Psychnol. J.* 3 101–112. 22954870

[B61] Riva G. Mantovani F. Capideville C. S. Preziosa A. Morganti F. Villani D. (2007). Affective interactions using virtual reality: the link between presence and emotions. *Cyberpsychol. Behav.* 10 45–56. 10.1089/cpb.2006.9993 17305448

[B62] Rizzo A. Parsons T. D. Lange B. Kenny P. Buckwalter J. G. Rothbaum B. (2011). Virtual reality goes to war: a brief review of the future of military behavioral healthcare. *J. Clin. Psychol. Med. Settings* 18 176–187. 10.1007/s10880-011-9247-2 21553133

[B63] Rizzo A. Reger G. Gahm G. Difede J. Rothbaum B. (2008). “Virtual reality exposure therapy for combat related PTSD,” in *Post-Traumatic Stress Disorder: Basic Science and Clinical Practice*, eds Shiromani P. Keane T. LeDoux J. (New York, NY: Springer Verlag).

[B64] Rodríguez A. Rey B. Clemente M. Wrzesien M. Alcañiz M. (2015a). Assessing brain activations associated with emotional regulation during virtual reality mood induction procedures. *Expert Syst. Appl.* 42 1699–1709. 10.1016/j.eswa.2014.10.006

[B65] Rodríguez A. Rey B. Vara M. D. Wrzesien M. Alcaniz M. Banos R. M. (2015b). A VR-based serious game for studying emotional regulation in adolescents. *IEEE Comput. Graph. Appl.* 35 65–73. 10.1109/MCG.2015.8 25666600

[B66] Rubin D. C. (2005). A basic-systems approach to autobiographical memory. *Curr. Dir. Psychol. Sci.* 14 79–83. 10.1111/j.0963-7214.2005.00339.x 25460237

[B67] Rubin D. C. Kozin M. (1984). Vivid memories. *Cognition* 16 81–95. 10.1016/0010-0277(84)90037-4 6540650

[B68] Saadatmand V. Rejeh N. Heravi-Karimooi M. Davood Tadrisi S. Zayeri F. Vaismoradi M. (2013). Effect of nature-based sounds’ intervention on agitation, anxiety, and stress in patients under mechanical ventilator support: a randomised controlled trial. *Int. J. Nurs. Stud.* 50 895–904. 10.1016/j.ijnurstu.2012.11.018 23245705

[B69] Serino S. Triberti S. Villani D. Cipresso P. Gaggioli A. Riva G. (2014). Toward a validation of cyber-interventions for stress disorders based on stress inoculation training: a systematic review. *Virtual Real.* 18 73–87. 10.1007/s10055-013-0237-6

[B70] Smith J. C. (1999). *ABC Relaxation Training: An Evidence-Based Approach.* New York, NY: Springer.

[B71] Triberti S. Barello S. (2016). The quest for engaging AmI: patient engagement and experience design tools to promote effective assisted living. *J. Biomed. Inform.* 63 150–156. 10.1016/j.jbi.2016.08.010 27515924

[B72] Tugade M. M. Fredrickson B. L. Barrett L. F. (2004). Psychological resilience and positive emotional granularity: examining the benefits of positive emotions on coping and health. *J. Pers.* 72 1161–1190. 10.1111/j.1467-6494.2004.00294.x 15509280 PMC1201429

[B73] Tulving E. (1985). Memory and consciousness. *Can. Psychol.* 26 1–12. 10.1037/h0080017

[B74] Tusek D. L. Cwynar R. E. (2000). Strategies for implementing a guided imagery program to enhance patient experience. *AACN Adv. Crit. Care* 11 68–76. 10.1097/00044067-200002000-00009 11040554

[B75] Vara M. D. Baños R. M. Rasal P. Rodríguez A. Rey B. Wrzesien M. (2016). A game for emotional regulation in adolescents: the (body) interface device matters. *Comput. Hum. Behav.* 57 267–273. 10.1016/j.chb.2015.12.033

[B76] Vempati R. P. Telles S. (2002). Yoga-based guided relaxation reduces sympathetic activity judged from baseline levels. *Psychol. Rep.* 90 487–494. 10.2466/pr0.2002.90.2.487 12061588

[B77] Villani D. Carissoli C. Triberti S. Marchetti A. Gilli G. Riva G. (2018). Videogames for emotion regulation: a systematic review. *Games Health J.* 7 85–99. 10.1089/g4h.2017.0108 29424555

[B78] Villani D. Riva F. Riva G. (2007). New technologies for relaxation: the role of presence. *Psychol. Assoc.* 14 260–274. 10.1037/1072-5245.14.3.260

[B79] Villani D. Rotasperti C. Cipresso P. Triberti S. Carissoli C. Riva G. (2017). *Assessing the Emotional State of Job Applicants Through a Virtual Reality Simulation: A Psycho-Physiological Study.* Cham: Springer, 119–126. 10.1007/978-3-319-49655-9_16

[B80] Witvliet C. Ludwig T. E. Vander Laan K. L. (2001). Granting forgiveness or harboring grudges: implications for emotion, physiology, and health. *Psychol. Sci.* 12 117–123. 10.1111/1467-9280.00320 11340919

[B500] Wright D. Gaskell G. (1992). “The construction and function of vivid memories,” in *Theoretical Perspectives on Autobiographical Memory*, Vol. 65 eds Conway M. A. Rubin D. C. Spinnler H. Wagenaar W. A. (Springer: Dordrecht) 275–292. 10.1007/978-94-015-7967-4_16

[B81] Wrzesien M. Rodríguez A. Rey B. Alcañiz M. Baños R. M. Vara M. D. (2015). How the physical similarity of avatars can influence the learning of emotion regulation strategies in teenagers. *Comput. Hum. Behav.* 43 101–111. 10.1016/j.chb.2014.09.024

